# Understanding adaptive gait in lower-limb amputees: insights from multivariate analyses

**DOI:** 10.1186/1743-0003-10-98

**Published:** 2013-08-16

**Authors:** John G Buckley, Alan R De Asha, Louise Johnson, Clive B Beggs

**Affiliations:** 1 Division of Medical Engineering, School of Engineering, Design & Technology, University of Bradford, Richmond Road, Bradford BD7 1DP, UK; 2 Division of Allied Health Professions, School of Health Studies, University of Bradford Bradford, BD7 1DP, UK

**Keywords:** Lower-limb amputee, Adaptive gait, Locomotion, Obstacle avoidance, Multivariate analyses

## Abstract

**Background:**

In this paper we use multivariate statistical techniques to gain insights into how adaptive gait involving obstacle crossing is regulated in lower-limb amputees compared to able-bodied controls, with the aim of identifying underlying characteristics that differ between the two groups and consequently highlighting gait deficits in the amputees.

**Methods:**

Eight unilateral trans-tibial amputees and twelve able-bodied controls completed adaptive gait trials involving negotiating various height obstacles; with amputees leading with their prosthetic limb. Spatiotemporal variables that are regularly used to quantify how gait is adapted when crossing obstacles were determined and subsequently analysed using multivariate statistical techniques.

**Results and discussion:**

There were fundamental differences in the adaptive gait between the two groups. Compared to controls, amputees had a reduced approach velocity, reduced foot placement distance before and after the obstacle and reduced foot clearance over it, and reduced lead-limb knee flexion during the step following crossing. Logistic regression analysis highlighted the variables that best distinguished between the gait of the two groups and multiple regression analysis (with approach velocity as a controlling factor) helped identify what gait adaptations were driving the differences seen in these variables. Getting closer to the obstacle before crossing it appeared to be a strategy to ensure the heel of the lead-limb foot passed over the obstacle prior to the foot being lowered to the ground. Despite adopting such a heel clearance strategy, the lead-foot was positioned closer to the obstacle following crossing, which was likely a result of a desire to attain a limb/foot angle and orientation at instant of landing that minimised loads on the residuum (as evidenced by the reduced lead-limb knee flexion during the step following crossing). These changes in foot placement meant the foot was in a different part of swing at point of crossing and this explains why foot clearance was considerably reduced in amputees.

**Conclusions:**

These results highlight that trans-tibial amputees use quite different gait adaptations to cross obstacles compared with controls (at least when leading with their prosthetic limb), indicating they are governed by different constraints; seemingly related to how they land on/load their prosthesis after crossing the obstacle.

## Background

In complex biodynamic systems, such as gait, many interrelated factors are at work. While some of these factors are known, others are poorly understood, with the result that underlying dynamics of the system may be misinterpreted. In particular, because of interconnectivity between variables, it is often difficult to assess the contribution that individual measured variables make to the overall behaviour of a system.

When analysing gait it is common practice to take multiple kinematic measurements in an attempt to capture the dynamics of a participant’s motion. Univariate analysis is then generally performed to assess the impact on the system as a whole of any group differences that exist or interventions that may have been made. While valid, this approach is limited, in so much that it yields little information about the connectivity of the system. Consequently, subtle interrelational changes between variables may go unnoticed, with the result that somewhat limited insights are gained, or in a worse-case scenario, erroneous conclusions may be reached.

In this paper we use multivariate statistical techniques to analyse the adaptive gait dynamics of lower-limb amputees and able-bodied controls, with the aim of identifying underlying gait characteristics that differ between the two groups. Any distinctions made arise purely from spatiotemporal data collected from individuals stepping over obstacles of varying heights. We chose obstacle crossing as it is a task that requires gait to be adapted during the approach to and step-over the obstacle. We predicted that the amputee participants would have different adaptive gait dynamics to those in able-bodied controls because of the constraints imposed by the prosthesis, and because it is known that they find obstacle crossing somewhat problematic [1,2]. We base our analyses on seven simple spatiotemporal variables (Table 1) that are regularly used to quantify how gait is adapted when crossing obstacles [3-9]. These variables were analysed using a number of techniques in an attempt to identify the ones that best distinguish the differences between the two groups. The overall aim of the study was to gain new insights in to the adaptive gait dynamics of amputees compared to able-bodied individuals and ultimately to identify underlying gait characteristics that differed between the two groups and consequently highlight gait deficits in the amputees.

**Table 1:** Comparison of adapted gait variables (mean and ± SD) between amputees and controls

		**High**	**Med**	**Low**	**Grp diff (p value)**
*toeC*	Amputees	63(25)	73(21)	77(22)	
	Controls	117(27)	130(28)	123(31)	0.0002*
*lead-1*	Amputees	−788(128)	−755(172)	−784(132)	
	Controls	−956(91)	−929(139)	−907(175)	0.008
*trail-1*	Amputees	−194(82)	−212(101)	−197(84)	
	Controls	−237(73)	−249(67)	−247(78)	0.23
*lead + 1*	Amputees	198(46)	192(62)	195(52)	
	Controls	263(77)	261(81)	242(73)	0.06
*heelC*	Amputees	44(28)	47(28)	62(27)	
	Controls	61(27)	73(23)	66(17)	0.15*^g-h^
*toeP-maxE*	Amputees	246(133)	260(181)	290(222)	
	Controls	73.7(166.4)	116.5(162.5)	269.5(183.9)	0.14^^g-h^
*kneeF*	Amputees	14(8)	14(9)	13(9)	
	Controls	20(7)	21(6)	21(5)	0.03
*vel-appr*	Amputees	0.97(0.11)	0.97(0.14)	1.0(0.11)	
	Controls	1.14(0.16)	1.18(0.14)	1.21(0.17)	0.001

All variables are measured in mm accept ‘*kneeF*’ which is degrees and ‘*walking speed*’ which is m/s.

‘Grp diff’ indicates group main effects determined by ANOVA. Significant obstacle height effects are indicated by *(p < 0.05) or ^(p < 0.001). Group-by-obstacle height interactions are indicated by g-h (p < 0.05).

## Methods

### Participants

Eight community-dwelling, physically active uni-lateral trans-tibial amputees (7 male, 1 female, mean age 46.2 ± 13.1 years; height 1.80 m ± 0.07 m; mean residual limb length 0.16 ± 0.02 m) took part in the study. All had undergone amputation due to trauma, infection or carcinoma at least two years prior to participation (mean 15.0 ± 12.3 years, range 5–36 years), regularly wore (at least six hours a day) and ambulated independently in their prosthesis (SIGAM score E or F, [10]). All had used their current prosthesis for at least three months and had not undergone rehabilitation in the previous six months. Twelve healthy able-bodied adult volunteers recruited from University staff acted as (age-matched) controls (8 male, 4 female, mean age 46.2 ± 8.1 years; height 1.73 ± 0.11 m). All participants were free from neurological, musculoskeletal (other than limb amputation) or cardiovascular disorders and were not on any medication that might interfere with balance, reaction time or co-ordination. Six amputees used a Multiflex, one a Flex-freedom, and one a Seattle Litefoot device. During data collection participants wore lycra shorts, a t-shirt (female) or were bare-chested (males), and comfortable flat-soled shoes. The tenets of the Declaration of Helsinki were observed and the experiment gained approval from the National Health Service (UK) Local Research Ethics Committee (Yorkshire and Humber), and Biomedical, Natural and Physical Sciences Research Ethics Panel of University of Bradford. All participants gave written informed consent and were asked to refrain from alcohol from the evening prior to testing.

### Obstacle crossing protocol

Data collection took place within a motion analysis laboratory with 8 m walkway. Participants were instructed to walk in a straight line across the laboratory at a self-selected comfortable speed and to step over an obstacle (height of 3, 7 or 10 cm; *low*, *medium*, *high*) placed in their travel path. Each obstacle (0.51 m wide, 0.005 m deep) was free-standing and would tip over easily if hit. Each participant’s starting position (approximately 2 m from the obstacle) was adjusted until they stepped over the obstacle consistently in a natural and comfortable manner. Participants were then given familiarisation trials (typically two) until they felt comfortable with the procedure. Each participant performed, in random order, three walking trials for each obstacle height. Amputees completed half the trials leading with the prosthetic and half with the intact limb (18 trials in total). Lead limb was block randomised and counter-balanced across amputee participants. None of the participants completed trials in which they avoided the obstacle and there were no trials in which a participant contacted the obstacle. The present study reports only prosthetic lead data (9 trials). Able-bodied volunteers always led with the same self-selected limb in all trials (n = 9).

### Movement analysis

Kinematic data were collected (100 Hz) using an eight-camera, motion analysis system (Vicon MX, Oxford, U.K.). Reflective markers (14 mm diameter, except on the feet where 7 mm diameter were used) were attached to each participant either directly onto skin, the lycra shorts or shoes (some of which were attached via elasticised bands) bilaterally to the following locations (or equivalent points on the prosthesis); superior aspect of the distal end of the 2nd toes, 2nd and 5th metatarsal heads, medial and lateral aspects of the midfoot, medial and lateral malleolus, posterior calcaneous, medial and lateral femoral epicondyles, greater trochanter, highest point of iliac crest, acromion, antero-lateral and postero-lateral aspects of the head. Markers were also placed on the xiphoid process, jugular notch, spinous processes of the 7th cervical and 8th thoracic vertebrae. Plate-mounted 4-marker clusters were attached to each thigh and shank and a skin-mounted 4-marker cluster was placed around the sacrum. Markers were also placed on the top of each obstacle. The anterior (toe) and posterior (heel) inferior shoe tips were retrospectively defined by determining their positions relative to the 2nd and 5th metatarsal head and 2nd toe markers, and calcaneal and malleoli markers respectively [11]. The marker set incorporated anatomical and tracking markers. Anatomical markers were located on bony landmarks or over joints and were removed after a static calibration trial, prior to the dynamic trials (44 markers remained during walking trials). Functional joint centres were created as virtual landmarks for all intact joints [12] including prosthetic limb knee joint. The prosthetic ankle joint was located on the mid-line of the prosthetic pylon at the same height as the functional joint centre on the intact ankle (see De Asha et al. 2013 for further details regarding data collection methodology [13]).

Trajectory data of each marker were labelled and then exported to Visual3D (C-Motion, Version 4.00.20, Germantown, MD, USA) for low-pass filtering (zero-lag Butterworth 6 Hz cut-off) and processing to define a 3-D linked nine-segment model of the participant. The three-dimensional co-ordinates of the obstacle, each foot’s heel and shoe tip, and the body centre of mass, along with prosthetic limb knee flexion (sagittal plane angular displacement), were exported (100 Hz) in ASCII format for further analysis.

The following parameters were determined:

Lead (*lead-1*) and trail (*trail-1*) foot-obstacle placement before the obstacle: the antero-posterior (A/P) horizontal distances between each toe-shoe tip (during ground contact) and the obstacle. Lead-foot placement beyond the obstacle (*lead + 1*): the A/P horizontal distance between the lead-limb heel (during ground contact) and the obstacle. Lead-limb toe and heel clearance (*toeC*, *heelC*): the vertical distance between the toe and heel shoe tips and the upper edge of the obstacle as each respective part of the foot crossed over the obstacle. Where the instant of maximum toe elevation occurred relative to the obstacle (*toeP-maxE*): the A/P distance between the shoe tip (toe) and the obstacle at instant of maximum toe elevation. Knee flexion during limb-loading (weight transfer onto lead limb) following crossing of obstacle (*kneeF*): the peak amount of knee flexion, relative to that determined during a standing calibration trial, during initial ground contact following completion of the crossing step. Average approach velocity (*vel-appr*): the average A/P velocity of the body centre of mass from when participant entered the motion-capture volume (approximately 2 m from obstacle) to point of lead-limb toe crossing.

### Data and statistical analysis

Because of corrupted or missing data some trial repetitions were omitted from the analysis (i.e. for the healthy controls, one at the high obstacle, and four at the medium obstacle were omitted; for the amputees, six repetitions at the high obstacle, two at the medium obstacle, and two at the low obstacle were omitted).

Each gait variable, averaged across repetition, was initially analysed using mixed design three (obstacle height) by two (group) repeated measures analysis of variance (ANOVA) with obstacle height as within subjects factor. Post-hoc analyses were performed using Tukey’s HSD. Analyses were undertaken using Statistica 5.5 for Windows (StatSoft Inc., Tulsa, USA).

In order to gain additional insights into the altered gait dynamics between the two groups, multivariate analyses were performed using the following analysis techniques:

(1) Logistic regression was used to identify the key variables that distinguish between the gait of the controls and the amputees.

(2) Partial correlation matrices (Pearson’s r correlation) were computed for the respective control and amputee cohorts, to identify changes in the interrelationships between the variables within the dataset.

Because we weren’t interested in the effects of obstacle height *per se*, the data analysed were either aggregated across obstacle height (logistic regression) or it was included as a controlling factor (partial correlation). In addition, as we were aware that amputees typically walk slower than their able-bodied counterparts [14,15] average approach velocity (*vel-appr*) was not included as part of the logistic regression analysis but it was included as a controlling factor in the correlational analyses.

Analyses were performed using a combination of Statistical Package for Social sciences (SPSS, IBM, Armonk, New York, USA) and in-house algorithms written in Matlab (Mathworks Inc., MA, USA).

Level of significance for all above statistical analyses was p < 0.05.

## Results

### Univariate analysis

Table 1 presents the results of the univariate analysis for the respective variables at the three obstacle heights. *vel-appr*, *toeC*, *lead-1*, *lead + 1*, and *kneeF* were all significantly reduced in amputees compared to able-bodied controls, while *trail-1* was similar between groups. (NB. Table 1 also indicates obstacle height effects). A significant group-by-obstacle height interaction (p = 0.035) indicated that *toeP-maxE* was increased in amputees compared to controls at the high and medium obstacle height, but there was no difference between groups at the low obstacle height. A significant group-by-obstacle height interaction (p = 0.04) indicated that *heelC* was reduced in the amputees compared to controls but only when crossing the medium height obstacle.

### Logistic regression analysis

Table 2 present the results of the logistic regression analysis. The variables *toeC*, *lead + 1, heelC,* and *kneeF* were statistically significant, indicating that any differences in the gait dynamics between the two cohorts are likely to be associated with these variables. Indeed, such was the ‘clear-cut’ nature of the logistic regression analysis, that sensitivity and specificity scores of 98.5% and 96.5%, respectively were achieved when data for all the obstacle heights were aggregated (Table 3).

**Table 2:** Results of logistic regression analysis: the significant variables that distinguish the gait of the controls and the amputees (data aggregated across obstacle heights)

**Variable**	**Beta**	**Standard error**	**Significance (p value)**
*toeC*	−0.197	0.058	0.001
*lead + 1*	0.060	0.018	0.001
*toeP-maxE*	−0.010	0.004	0.014
*kneeF*	−0.349	0.107	0.001
*heelC*	0.123	0.047	0.009

**Table 3:** Results of logistic regression analysis: sensitivity and specificity of the variables for all participants, all obstacle heights

**Obstacle height**	**True positives**	**False negatives**	**True negatives**	**False positives**	**Sensitivity**	**Specificity**	**Significance (p value)**
All	65	1	82	3	98.5%	96.5%	<0.0001^
Low	22	0	30	0	100.0%	100.0%	<0.0001^
Medium	22	1	24	2	95.7%	92.3%	<0.0001^
High	21	0	29	0	100.0%	100.0%	<0.0001^

^Significance determined using Chi squared test applied only to true positives and false negatives.

### Partial correlation analysis

The results of the partial correlation analysis are presented in Tables 4 and 5, which shows the correlation coefficients, r, and p values for the respective variables, when controlling for obstacle height and approach velocity. The results are compiled from analysis of the complete dataset containing 9 trials per participant, i.e. data from a total of 180 trials. Comparison between these tables reveals marked differences in some of the relationships between the variables in the amputee group compared with the control group. For example, in the controls, there is no correlation between *toeC* and *toeP-maxE* (r = 0.09; p = 0.43), whereas in the amputees, there is a relatively strong negative correlation between these two variables (r = −0.50; p < 0.0001). Likewise the relationship between *heelC* and *toeP-maxE* is very weak in the control group (r = 0.06; p = 0.57), whereas it is relatively strong in the amputees (r = 0.48; p = 0.0001). Conversely, the correlation between *kneeF* and *lead + 1* is relatively strong in the controls (r = 0.45; p < 0.0001) but weak in the amputees (r = −0.22; p = 0.09). Finally, the relationship between *kneeF* and *trail-1* is markedly different between the two cohorts. In the amputees there is no correlation evident (r = −0.17 p = 0.17), whereas in the controls there is a strong correlation between these two variables (r = 0.67; p <0.001). The table also shows that in both groups there is strong correlation between the variables; *trail-1* and *lead + 1*, *lead + 1* and *toePmaxE*, and *lead + 1* and *heelC*.

**Table 4:** Partial correlation matrices (r (2-tailed p value)) for controls, controlling for obstacle height

	**toeC**	**lead-1**	**trail-1**	**lead + 1**	**toeP-maxE**	**kneeF**	**heelC**
**toeC**		0.2754	0.3133	0.0502	0.0875	0.1650	0.4245
		(0.0118)	(0.0039)	(0.6525)	(0.4314)	(0.1361)	(0.0001)
**lead-1**	0.2754		0.4341	0.0132	−0.2966	0.2191	−0.1405
	(0.0118)		(0.0000)	(0.9058)	(0.0065)	(0.0466)	(0.2052)
**trail-1**	0.3133	0.4341		0.5088	0.1005	0.6650	−0.4208
	(0.0039)	(0.0000)		(0.0000)	(0.3660)	(0.0000)	(0.0001)
**lead + 1**	0.0502	0.0132	0.5088		0.5104	0.4458	−0.4259
	(0.6525)	(0.9058)	(0.0000)		(0.0000)	(0.0000)	(0.0001)
**toeP-maxE**	0.0875	−0.2966	0.1005	0.5104		0.0587	0.0630
	(0.4314)	(0.0065)	(0.3660)	(0.0000)		(0.5981)	(0.5716)
**kneeF**	0.1650	0.2191	0.6650	0.4458	0.0587		−0.3518
	(0.1361)	(0.0466)	(0.0000)	(0.0000)	(0.5981)		(0.0011)
**heelC**	0.4245	−0.1405	−0.4208	−0.4259	0.0630	−0.3518	
	(0.0001)	(0.2052)	(0.0001)	(0.0001)	(0.5716)	(0.0011)	

**Table 5:** Partial correlation matrices (r (2-tailed p value)) for amputees, controlling for obstacle height

	**toeC**	**lead-1**	**trail-1**	**lead + 1**	**toeP-maxE**	**kneeF**	**heelC**
**toeC**		0.4283	0.3882	−0.3340	−0.4972	−0.0652	0.7031
		(0.0004)	(0.0015)	(0.0070)	(0.0000)	(0.6090)	(0.0000)
**lead-1**	0.4283		0.2913	−0.0501	−0.2952	0.0677	0.1794
	(0.0004)		(0.0195)	(0.6940)	(0.0179)	(0.5949)	(0.1560)
**trail-1**	0.3882	0.2913		0.4601	−0.1190	−0.1734	−0.0920
	(0.0015)	(0.0195)		(0.0001)	(0.3488)	(0.1705)	(0.4696)
**lead + 1**	−0.3340	−0.0501	0.4601		0.5716	−0.2156	−0.6369
	(0.0070)	(0.6940)	(0.0001)		(0.0000)	(0.0871)	(0.0000)
**toeP-maxE**	−0.4972	−0.2952	−0.1190	0.5716		−0.1923	−0.4768
	(0.0000)	(0.0179)	(0.3488)	(0.0000)		(0.1279)	(0.0001)
**kneeF**	−0.0652	0.0677	−0.1734	−0.2156	−0.1923		−0.1176
	(0.6090)	(0.5949)	(0.1705)	(0.0871)	(0.1279)		(0.3546)
**heelC**	0.7031	0.1794	−0.0920	−0.6369	−0.4768	−0.1176	
	(0.0000)	(0.1560)	(0.4696)	(0.0000)	(0.0001)	(0.3546)	

## Discussion

The univariate (ANOVA) analysis indicated significant group differences in *vel-appr*, *toeC*, *lead-1*, *kneeF*, a group-by-obstacle height difference in *toeP-maxE* (Table 1), and a group difference trend in *lead + 1*. These differences indicate that the amputees had a reduced walking velocity, were closer to the obstacle prior to, during, and following crossing it (i.e. lead foot placement before and after the obstacle and clearance over it were reduced for amputees compared to controls), and had reduced knee flexion during loading response (transfer of body weight onto the leading limb) for the step immediately after crossing the obstacle. The only variable that was increased in the amputees was the horizontal position of the toe relative to the obstacle at instant of maximum toe elevation (i.e. *toeP-maxE*); though only significantly when crossing the medium and high obstacles. The larger *toeP-maxE* values in the amputees indicate that the point of maximum toe elevation was further beyond the obstacle in the amputees compared to the controls, and this is likely linked to the group differences found in the foot placements when approaching the obstacle. However, what is not clear from this analysis is ‘what’ is driving ‘what’? For example, do amputees focus on where and how high the lead-limb foot should be at the point of crossing, and/or point of maximum elevation, and then alter their foot placements to facilitate this? Or, do they focus more on foot placement before/after the obstacle and as a consequence, where in swing the foot is when it crosses the obstacle and/or at point of maximum elevation simply becomes a ‘by-product’ of foot placement? Alternatively, both foot placement and lead-limb (foot) trajectory could be controlled independently and thus one is not necessarily a consequence of the other. Our aim was therefore to analyze the data in greater depth so as to better understand what is driving the key differences between the gait dynamics of the controls and the amputees.

**Figure 1:**
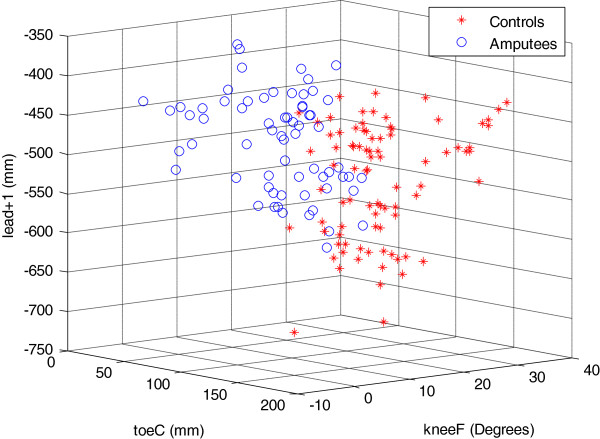
**3D plot of *lead + 1*, *toeC *and *kneeF *plotted against each other for the two groups.** Data shown are from all trials for all participants.

The results of the logistic regression analysis support those of the ANOVA, and indicate the variables *toeC*, *heelC*, *toeP-maxE*, *lead + 1*, and *kneeF*, all contributed significantly to the differences in gait between the two cohorts. To emphasise that the differences in gait between the two cohorts were associated with these highlighted variables, we constructed a 3D plot using three out of the four variables with the highest Beta values (Table 3) plotted against each other: *heelC* was not included because of its semblance to *toeC*. This figure (Figure 1) highlights a clear distinction between the two cohorts, and thus suggests fundamental differences in the adaptive gait between the two groups.

**Figure 2:**
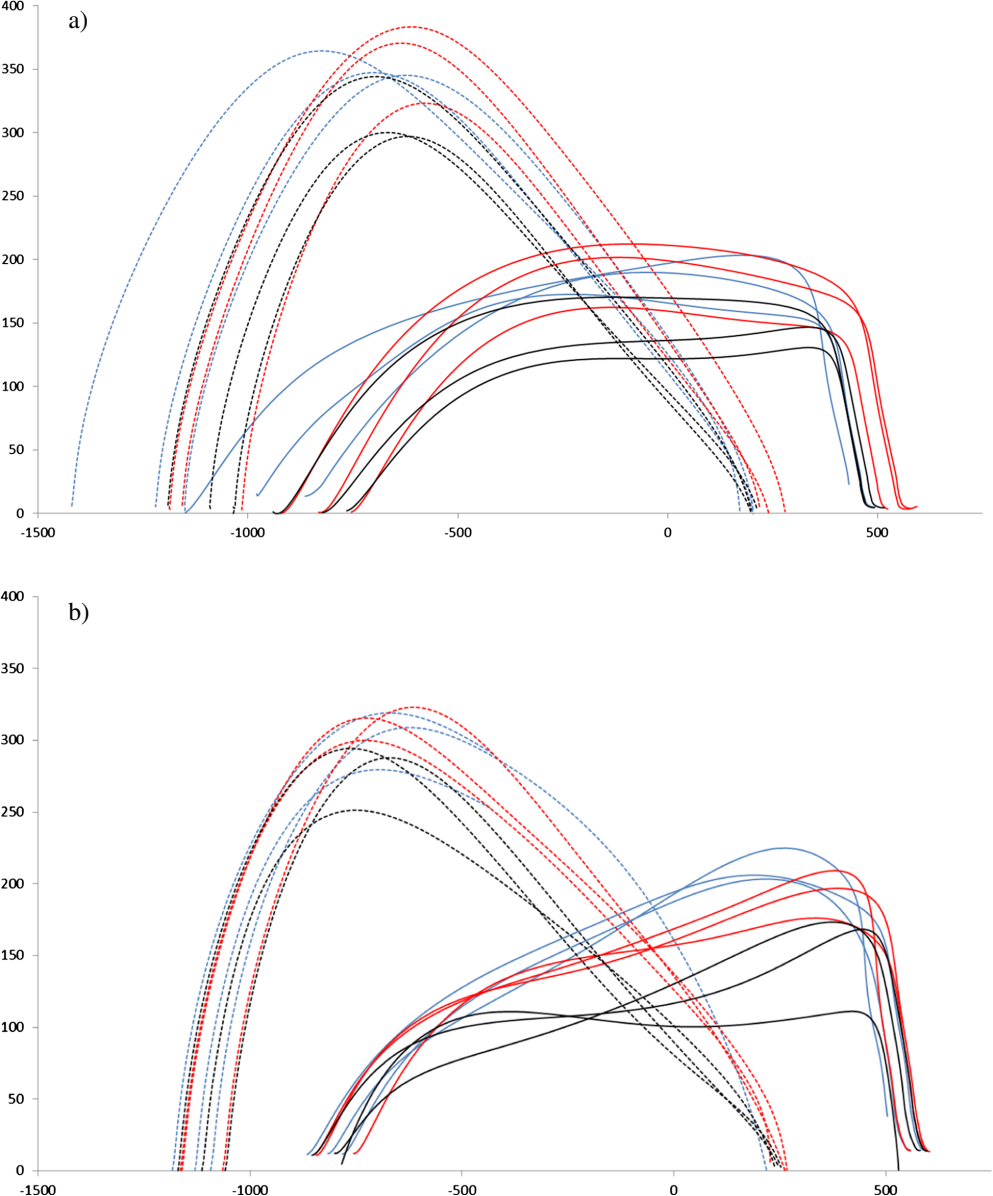
**Lead-limb toe (solid lines) and heel (dotted lines) trajectories over the obstacle for the 9 obstacle crossing trials for one control (a) and one amputee (b) participant.** The zero reference point indicates the obstacle location. Black, red and blue lines indicate low, medium and high obstacle heights respectively.

The results of the correlation analysis (Tables 4 and 5) revealed marked differences between the two cohorts in the correlations between, or with, the key gait variables (as identified by the logistic regression analysis and/or ANOVA analysis) which, in part, may help to explain what the causes of the group differences in adaptive gait are. Specifically, large differences exist between the two cohorts in the correlations between *toeC/heelC* and *toeP-maxE*, and between *trail-1/lead + 1* and *kneeF*. It is important to highlight that these differences were revealed after controlling for walking speed (i.e. *vel-appr* was included as a controlling factor). The relationships between *trail-1*/*lead + 1* and *kneeF* are weakly negative in the amputees and strongly positive in the controls. When combined the variables *trail-1* and *lead + 1* indicate the length of the crossing step. The significant positive correlation in controls (between these variables and the variable *kneeF*) indicates that as crossing step length increased there was an increase in knee flexion. The weak and non-significant correlation in amputees (between the same variables) indicates there was no relationship between crossing step length and knee flexion. This suggests they limited the amount of knee flexion occurring during loading response irrespective of where they placed their feet before and after the obstacle. This may be reflective of a strategy to reduce the moment (loading) at the residual knee and/or end of residuum which previous research has shown is something amputees tend to do during over-ground gait [14,15]. Regarding the relationships between *toeC*/*heelC* and *toeP-maxE* in the controls there are no relationships between these variables, while in the amputees the relationships between these variables are strongly negatively. The variable *toeP-maxE* indicates the horizontal position of the toe relative to the obstacle at the point of maximum toe elevation. Thus in the controls, foot clearance appears to be unrelated to the elevation trajectory of the foot; which seems somewhat nonsensical. The lack of correlation is more likely related to the fact that *toeP-maxE* in the controls varied between being positive or negative (Table 1; note the high variability relative to the mean), and thus the point of toe and heel crossing (and thus clearance) occurred either on the ‘upward’ part of the foot elevation trajectory or on the ‘downward’ part. In contrast, in the amputees *toeP-maxE* was consistently positive (Table 1; note the low variability relative to the mean), indicating that the point of toe and heel crossing (and thus clearance) consistently occurred on the ‘upward’ part of the foot elevation trajectory. The negative correlation between *toeC*/*heelC* and *toeP-maxE* indicates that as foot clearance increased *toeP-maxE* was reduced. This indicates that toe and heel clearances were a function of where in the ‘upward’ trajectory the foot was when it crossed the obstacle (which seems logical). Figure 2 presents lead-limb toe and heel trajectories for all nine obstacle crossing trials for one amputee and one able-bodied control, and the figure helps highlight the differences between groups in foot clearance strategy over the obstacle (as described above). The figure highlights that the toe elevation trajectory in the amputee had a steady incline (increase) from lead-limb foot-off up to point of maximum toe elevation (*toeP-maxE*), whereas in the able-bodied control toe elevation increased more sharply and then had a period of being at a relatively constant height.

Although toe clearance (*toeC*) is the most widely reported variable in previous studies investigating adaptive gait [3-9]; at least one of these studies indicated foot placement before the obstacle was key to crossing success [5]. The particular study in question also reported that in successful obstacle crossing trials, the point at which maximum toe elevation occurred was after the obstacle, while in unsuccessful trials it occurred before the obstacle [5]. In the present study, the high variability in *toeP-maxE* (relative to mean, Table 1) in controls, particularly when crossing the medium and high obstacles, indicates that in some participants and/or in some trials, the point of maximum toe elevation occurred before the obstacle. This indicates that having the point of maximum toe elevation occur after the obstacle is not a requirement for successful obstacle negotiation. In all amputees, the instant of maximum toe elevation consistently occurred after the obstacle (as evidenced by the reduced SD relative to mean, Table 1). Our results thus suggest amputees gave increased importance to *toeP-maxE*. This may be why *toePmaxE* was correlated with a number of variables in the amputee group (*toeC*, *heelC*, *lead + 1*) but only linked with one other variable in the controls (*lead + 1*, Tables 4 and 5). Having *toeP-maxE* consistently occur someway after the obstacle would minimize the chances of an inadvertent heel contact with the obstacle as the foot is lowered to the ground to complete the crossing step. It is noteworthy that the average *toeP-maxE* value in amputees (across obstacle heights) was 265 mm (Table 1), which approaches the length of the average sized foot. Collectively the above findings suggest that the amputee participants attended to how and when their heel (entire foot) passed over the obstacle. Despite the increase in mean *toeP-maxE* in amputees, *lead + 1* was reduced for amputees compared to controls. This foot (toe, heel) clearance/elevation strategy was likely related to the lack of ankle dorsi/plantar –flexion available and/or from a desire to attain a limb/foot angle and orientation at instant of landing (following crossing the obstacle) that minimized loads on the residuum. The fact that toe and heel clearance was reduced in amputees compared to controls, suggests that minimising loads on the residuum may be a more important driver of adaptive gait in amputees than maximising foot clearance safety margins.

Previous research has shown amputees find crossing obstacles problematic [1,2,16] particularly so when negotiating suddenly appearing obstacles under reduced time pressure [1,16]. A higher failure rate in this task compared to able-bodied controls was associated with them having decreased muscle activity response amplitudes; such that they were less able to adjust their stepping pattern irrespective of which limb they were leading with [1,16]. The findings of the present study would appear to corroborate these previous findings, as they indicate amputees are governed by different constraints; seemingly related to how they land on/load their prosthesis during the step following obstacle crossing. This may mean the gait of amputees is less adaptable than the gait of able-bodied controls. Note the lack of variability in foot position distance in the amputee compared to control in Figure 2. The accompanying reduction in toe and heal clearance indicates that the margins of safety are reduced in amputees compared to controls. Such reduced margins of safety may help explain why amputees have an increased risk of falling compared with age-matched able-bodied individuals [2,17]. In the study by Hofstad and colleagues [16] it was suggested that the former previous studies investigating obstacle crossing in unilateral trans-tibial amputees [18,19] were unable to reveal gait deficits in the amputee participants because the tasks employed were not difficult enough. These studies did however suggest that amputees crossed obstacles in a more cautious manner. The findings of the present study, which were gained using a similar task to those previously used by Hill and colleagues [18,19], would suggest that it wasn’t necessarily the tasks formerly used that were limited but rather it was the analyses used that were limited.

A limitation of the study is that we did not compare how amputees cross obstacles when leading with their intact limb, or indeed evaluate which limb they prefer to cross obstacles leading with. We only present data for when leading with the prosthetic limb because previous research has shown that this is the limb the majority of trans-tibial amputees prefer to lead with when crossing obstacles [20], and we chose not to compare leading between prosthetic and intact limb lead as we believed this would detract from the main message of the study. Another limitation is the sample size of 8 trans-tibial amputees. Such a sample size is not untypical for studies involving lower-limb amputees; however, given the somewhat limited sample size, we believe the present study’s results should be viewed as preliminary. Finally, as expected, approach velocity (walking speed) was significantly reduced in amputees compared to controls, and this reduction may have contributed to the differences between the groups observed in foot placement distance or lead-limb knee flexion for the step immediately following crossing. However, the variables that were found to differ between the groups using ANOVA analysis or logistic regression analysis were also the ones highlighted by the partial correlational analyses as being the variables that differed between groups in their relationships with other variables. And importantly, these differences were found even though approach velocity was used as a controlling factor. This suggests that the differences seen between groups were not simply a result of the differences in walking speed.

## Conclusions

The findings of the present study indicate that when trans-tibial amputee participants negotiate different height obstacles leading with their prosthetic limb, they adapt their gait quite differently to the way able-bodied controls adapt theirs. When compared to controls, amputees had a reduced walking velocity, got much closer to the obstacle prior to, during and following crossing it, and had reduced lead-limb knee flexion during the step following crossing. Getting closer to the obstacle before crossing it appeared to be a strategy to ensure the heel of the lead-limb passed over the obstacle prior to the foot being lowered to the ground. Despite adopting such a heel clearance strategy, the lead-foot was positioned closer to the obstacle following crossing, which was likely a result of a desire to attain a limb/foot angle and orientation at instant of landing that minimised loads on the residuum (which would explain the reduced lead-limb knee flexion evident for the step following crossing). The differences in foot position distances before and after the obstacle meant the foot was in a different part of swing at point of crossing, and this explains why amputees had considerably reduced foot (toe and heel) clearance over the obstacle. These results suggest that the gait adaptations used by amputees to cross different height obstacles are governed by different constraints when compared to able-bodied controls: seemingly related to how they land on/load their prosthesis after crossing the obstacle.

## Competing interests

The authors declare that they have no competing interests.

## Authors’ contributions

JB conceived the design of the study, oversaw motion capture data collection, performed the univariate statistical analysis, and helped to draft the manuscript. ADA and LJ helped define the study’s protocol, undertook data collection and processing, and provided critical comment during drafting of the manuscript. CB helped conceive the study, performed the multivariate statistical analyses, and helped to draft the manuscript. All authors read and approved the final manuscript.
